# ATHLETIC: An exoskeleton countermeasure exercise device for resistive and plyometric training in deep‐space missions

**DOI:** 10.1113/EP092263

**Published:** 2025-03-20

**Authors:** Jonas Böcker, Jochen Zange, Torsten Siedel, Guillaume Fau, Sebastian Langner, Thomas Krueger, Jörn Rittweger

**Affiliations:** ^1^ Institute of Aerospace Medicine German Aerospace Center Cologne Germany; ^2^ Space Applications Services Sint‐Stevens‐Woluwe Belgium; ^3^ deuter Sport GmbH Gersthofen Germany; ^4^ European Space Agency ESTEC Noordwijk The Netherlands; ^5^ Department of Pediatrics and Adolescent Medicine University Hospital Cologne Cologne Germany

**Keywords:** constant force mechanism, exoskeleton, low energy costs, microgravity, plyometric training, resistive training

## Abstract

Prolonged exposure to weightlessness leads to loss of muscle and bone mass. Therefore, astronauts on board the International Space Station (ISS) currently perform mandatory daily exercises. ISS missions usually last 6 months, and future missions will become significantly longer when going, for example, to Mars. To that purpose, an exoskeleton‐based exercise device, called ATHLETIC, was developed. The functionality and relevance of this device was evaluated in this study. Ten participants performed resistance exercises (squats) and plyometric exercises (countermovement jumps, reactive hops). Results showed that all participants were technically able to perform the intended exercises on ATHLETIC, albeit with reduced loading as compared to the reference exercises. This resulted in less mechanical performance and muscle activity. Due to the unfamiliar horizontal training axis, some participants had difficulty performing the movements correctly. Follow‐up studies are required testing, whether an adequate number of practicing sessions could enable persons to approach the performances of reference measurements, and whether further improvements of the device are needed to improve the exercise performance.

## Abstract (highlights)

- **What is the central question of the study?**
  A new type of training device (called ATHLETIC) was tested for functionality for complex plyometric and high‐intensive resistance exercises: are the planned exercises generally possible on the new training device and to what extent do the exercises performed on the device differ from reference exercises?
- **What is the main finding and its importance?**
  Squatting on the ATHLETIC device was technically possible, albeit with reduced loading as compared to reference exercises. Thus, although participants were able to jump and even hop, reactive muscle contractions were not possible as indicated, for example, by the long ground contact times.

## 1 INTRODUCTION

The daily resistance and endurance training during space missions onboard the International Space Station (ISS) partly maintains the physical fitness of the astronauts during long‐duration missions. However, there is still a sizeable loss of muscle and bone mass and loss of strength (Narici & de Boer, 2011; Pavy‐Le Traon et al., 2007). In addition, despite high daily training volume, post‐flight increases in fitness have never been observed (English et al., 2020; Fitts et al., 2010; Hargens et al., 2013; Rittweger et al., 2018). During exercise on Earth, a person operates with their own body weight generated by gravity, which acts directly on every segments of the body. In weightlessness, however, a simulated body weight or any additional loads can only be applied to the body from outside elements such as bars connected to external load generators like vacuum cylinders, for example the advanced resistive exercise device (ARED), or from a harness, as with the treadmill with vibration‐isolation stabilization system (TVIS). But the exclusive application of external forces can lead to discomfort from the harness (Gopalakrishnan et al., 2010). As a result, the astronauts on board the ISS generally train with significantly lower forces than they would on Earth (Cavanagh et al., 2010; Genc et al., 2010; Gopalakrishnan et al., 2010; Rittweger et al., 2018). It has been shown that these insufficient peak forces during strength training cannot be compensated by a higher training volume, which explains the limited success of training on board the ISS to this day (English et al., 2020; Rittweger et al., 2018).

In addition, the physiology of the human body seems to be specifically adapted to bouncing movements, like running and hopping. During such activities, peak forces are attained in bouts of 200–300 ms, which matches with the duration of short‐term monosynaptic reflexes, and also with the resonance frequency related to leg stiffness (Hobara et al., 2007). Such bouncing movements are characterised by consecutive muscle stretch and shortening (Komi, 2000). They are thought to support both storage and recovery of elastic energy. Because stretch‐shortening cycles involve elongation of activated muscles during the stretch phase, such exercises are also referred to as ‘plyometric’ exercises.

Plyometric training or so‐called impact training programmes have been proven as effective countermeasures against the loss of muscle and bone mass during bed rest studies. This was demonstrated during resistance training with whole‐body vibration (Blottner et al., 2006; Rittweger et al., 2010), flywheel training (Belavy et al., 2017; Rittweger et al., 2005) and combined jumping and strength training on a horizontal jumping sledge (Kramer et al., 2018, 2017; Ritzmann et al., 2018).

Therefore, optimal countermeasure and exercise devices for astronauts should enable both strength and endurance training at sufficiently high physical loads. However, due to the strict limitations on power resources inside a space vehicle like a Moon orbiter or a spaceship travelling to Mars, the high forces and power levels required for such exercising persons cannot be produced directly by high energy consuming motors or pumps. Noise and heat problems will share the same limitations. Similarly, the available volume for theses training devices will likely be more restricted compared to ones on board the ISS.

Based on these requirements, an exoskeleton device, based on pseudo‐anthropometric kinematics, was developed as a novel training device enabling resistive and plyometric training. The objective of this device was to combine a passive exoskeleton system with a pair of constant force generating mechanisms and inertia adjustable modules that will allow for natural hip, knee and foot motion during exercises with independently or synchronously operating legs. In general, the aim of this study was to validate the functionality of that novel countermeasure device, divided into the primary aim of examining whether the planned exercises are generally possible on the new training device and the secondary aim of gaining initial insights into the extent to which the exercises performed on the device differ from reference exercises. We focused on squatting as an example for a complex, coordinative challenging resistive exercise as well as on countermovement jumps (CMJ) and reactive forefoot hopping (HOP) as plyometric exercises.

## 2 METHODS

### 2.1 Participants and ethical approval

Ten participants (four male, six female) were included in this study. The participants were 176.6 cm (SD 11.2 cm) tall and the height ranged from 158 to 193 cm. The mean body mass was 73.8 kg (SD 13.2 kg) and ranged from 53 to 93 kg.

All participants were recreationally physically active. Applicants were excluded from study when they reported a history of injuries, disorders or disabilities that would limit the sportive performance, especially resistance and plyometric training.

Participants were fully acquainted with the experimental approach and provided a written informed consent prior to their participation. Study approval was issued on 20 December 2019 by the North Rhine Medical Association's Ethics Committee (*Ethikkommission der Ärztekammer Nordrhein*, Düsseldorf, Germany, approval no. 2019369). The study conformed to the standards set by the *Declaration of Helsinki*, except for registration in a database.

### 2.2 The ATHLETIC project

Within the ATHLETIC project (AstronauT HeaLth EnhancemenT Integrated Countermeasure; ESA Contract No. AO/1‐9473/18/NL/RA), an exoskeleton training device was developed by Space Applications Services (Zaventem, Belgium) in cooperation with the German Aerospace Center (DLR, Cologne, Germany) and deuter Sport GmbH (Gersthofen, Germany). In the first design iterations, the prototype was tested for fitting, ergonomics and comfort with none to moderate load forces. In this publication, we report the clinical testing of the final prototype, which was tested under the respective maximum load that a participant could handle alone in the exercise mode. The study took place from February till April 2023 at the Institute of Aerospace Medicine of the German Aerospace Center in Cologne, Germany. Each participant performed the reference exercise first directly followed by the exercise testing on ATHLETIC. The entire duration of testing was about 2 h and testing took place either in the morning or in the afternoon. As the tests concerned functionality of the device, the diet was not standardised before the respective tests.

### 2.3 The ATHLETIC device

The technical properties of the ATHLETIC device including the functions of the constant force mechanism, the inertia module and the telescopic kinematics have been previously published (Siedel et al., 2022). Here, an overview of the construction and function of the device is provided to support understanding of the exercises and testing.

**FIGURE 1:**
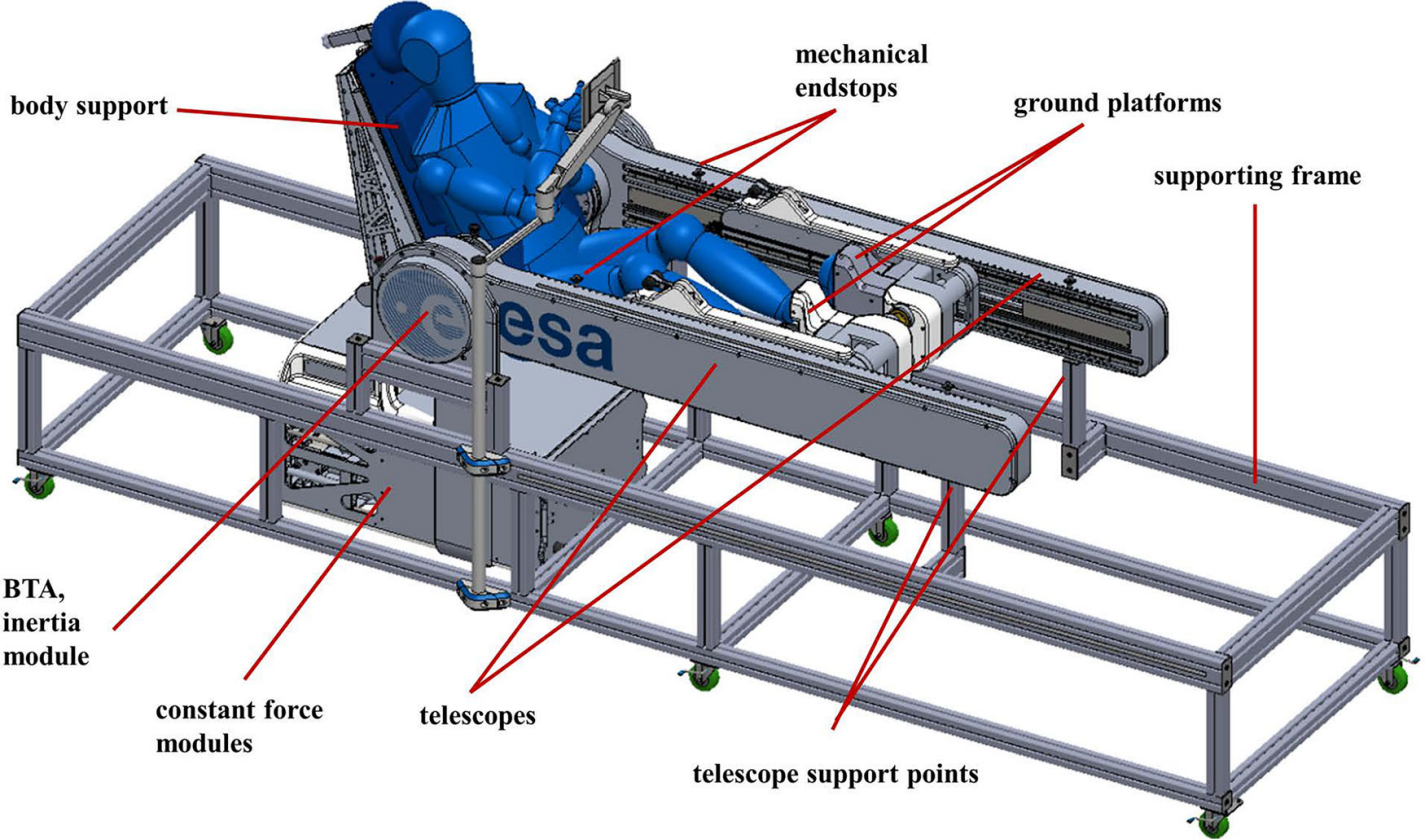
The ATHLETIC device carried by a supporting frame (MGSE: mechanical ground support equipment). The device is carried at the BTA (body support – telescope axis) and at the ends of the telescopes.

**FIGURE 2:**
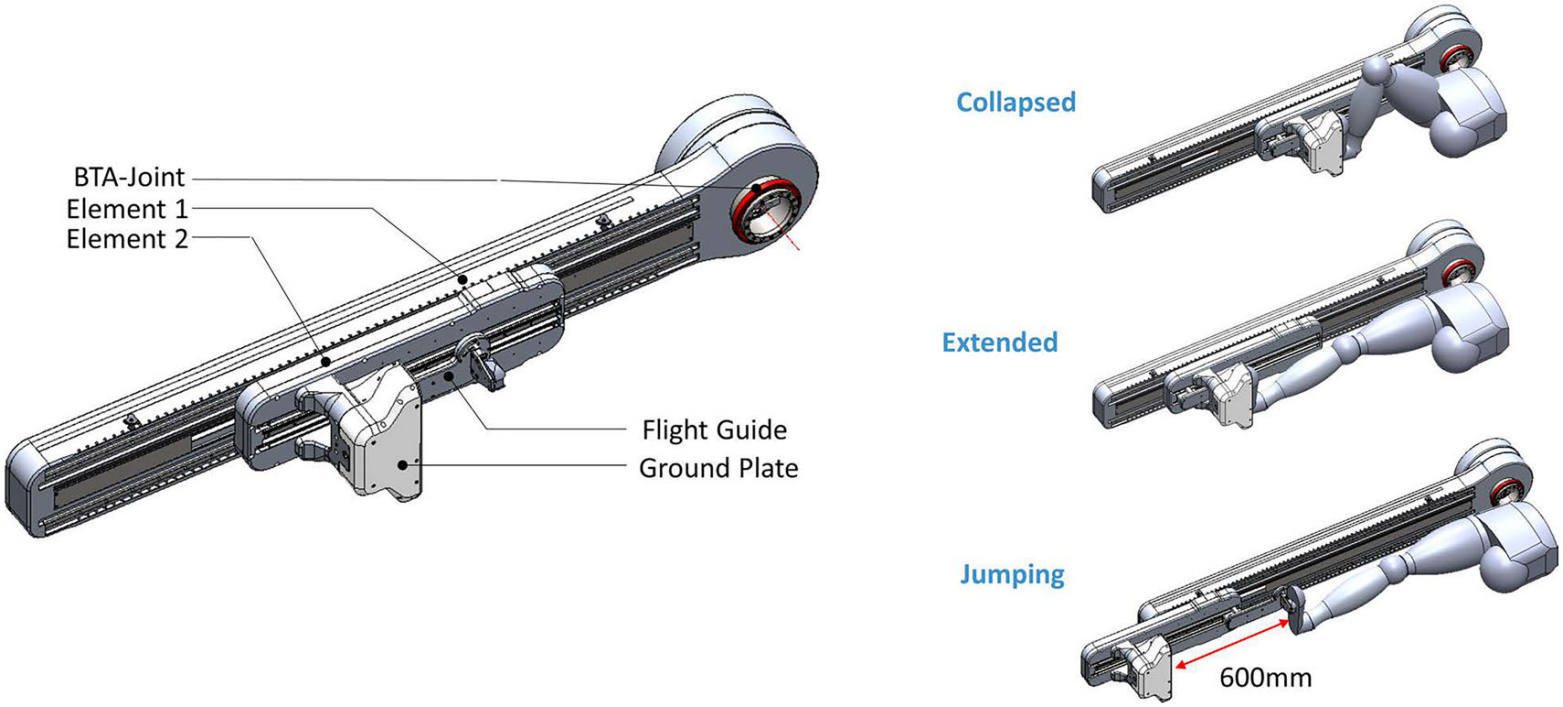
The elements and function of a telescope of ATHLETIC.

During the study, the participants were placed in a horizontal supine position on the ATHLETIC device (Figure 1). This posture simulated microgravity by unloading the body length axis from Earth gravity. The position of the head and trunk was supported by a surface for lying on that could rotate up‐ and downwards. The participants were fixed in position and prevented from headwards motion by a customized backpack harness that transferred the loads applied by the ATHLETIC device. The backpack harness held the participants at their shoulders and at the pelvis. A downwards motion of the participant, especially in sitting position, was prevented by a bicycle saddle. The central rotation axis of the upper frame (body torso axis – BTA) was individually positioned under the body's centre of gravity so that each participant could move the body without needing weight compensation through trunk muscle contraction. During squats or CMJ, the participant could move the body freely around this axis. During HOP, the BTA was fixed in a horizontal position. A participant could move the legs forwards and backwards either individually or in a coupled mode. However, in this study the exoskeleton telescopic legs only operated horizontally, because of their weight on Earth. During exercise, participants wore biking shoes that were attached to a click system along the telescopic ground platforms. The two feet elements could move independently or be mechanically linked, which allowed the participant either an independent, for example, side alternating foot motion, or a combined two leg action. A pair of constant force mechanisms transferred the load via the telescopes to their corresponding foot platforms, simulating an artificial gravity loading. The constant force was adjustable between 25 and 150 kilopond (kp) (1472 N) on each side. Additionally, a pair of inertia modules transferred a speed‐dependent inertial force to the corresponding side of the foot platform. In each module, the inertial force was generated by a pair of counter‐rotating masses, which were located around the BTA. Each rotating inertia system generated the forces corresponding with the inertial behaviour of an adjustable, constant mass between 29 and 43 kg. Each foot platform integrated a secondary telescope segment used as a flight guide to allow the foot to separate freely from the foot platform by up to 60 cm with low friction. During jumps, this segment carried the foot and guided it back to the platform during landing (Figure 2).

In preparation for an exercise session, the fixing in position of the participant on the body support, the belts of the backpack, the saddle position and the location of the rotation axis required individual manual adjustment. On both telescopes, individually positioned mechanical end stops limited the range of motion towards the participant preventing any injury. The feet were mounted to the platform when the force generating modules were in end stop position. The constant forces and the inertial mass could be individually adjusted by the supervising team using the provided graphical user interface (GUI). The ATHLETIC device measured and recorded at 1 kHz sampling rate the adjusted constant force, the current ground reaction force at the platform, the position of the platform on the telescope and the distance between the foot and the platform during a jump. In this study, the records from ATHLETIC were processed using custom‐made software based on the programming language R‐4.3.2 within RStudio 2023.12.0 Build 369.

### 2.4 Exercise preparation

Participants entered the ATHLETIC device wearing the provided biking shoes and their personal sport clothing. The support frame was tilted to 80° to help the participants to sit on the saddle. Both foot platforms were held in position by the mechanical end stops, which the participants could reach with their feet with moderately flexed knees. The participant fixed both feet by clicking the biking shoes into the holder on the fully retracted flight guide. Participants were further harnessed by the belts of the backpack construction on the body support. After donning, the body support was turned to a horizontal angle. Then the body support was horizontally moved head‐ and foot‐wards, up‐ and downwards until its centre of rotation was located below the centre of gravity of the participant's trunk so that the participant could rotate the trunk up and down without weight‐bearing by contracting the trunk muscles. Before starting with an exercise, the loads of the constant force mechanisms and mass inertia modules were adjusted while the foot platforms were held by the end stops and the knees were still unloaded in a flexed position. All exercises started with an active knee extension. When the load provided by the constant force mechanism was too high for this initial knee extension, it was stepwise adjusted to a lower value until the participant could move the platform. In consequence, a participant was only loaded with forces they could actively handle. The ATHLETIC system can only store and release mechanical energy that has initially been generated by the participant.

### 2.5 Resistive exercise: Squatting

On ATHLETIC, we tested squatting, which was compared with squatting on the ground as reference. The reference exercises were performed in a power rack. The participants carried an extra load of 50% body weight (% BW) applied on the shoulders by a barbell. This weight was chosen for a safe, medium‐intensity strength training programme of which all participants should be able to perform several repetitions without exhaustion. For squatting on the ATHLETIC device, we also aimed at exercising with a sum constant force equal to the individual 150% BW. However, if on ATHLETIC a participant could not correctly perform the exercise at 150% BW, the given load on ATHLETIC was lowered to the highest possible value at which it could be performed.

**FIGURE 3:**
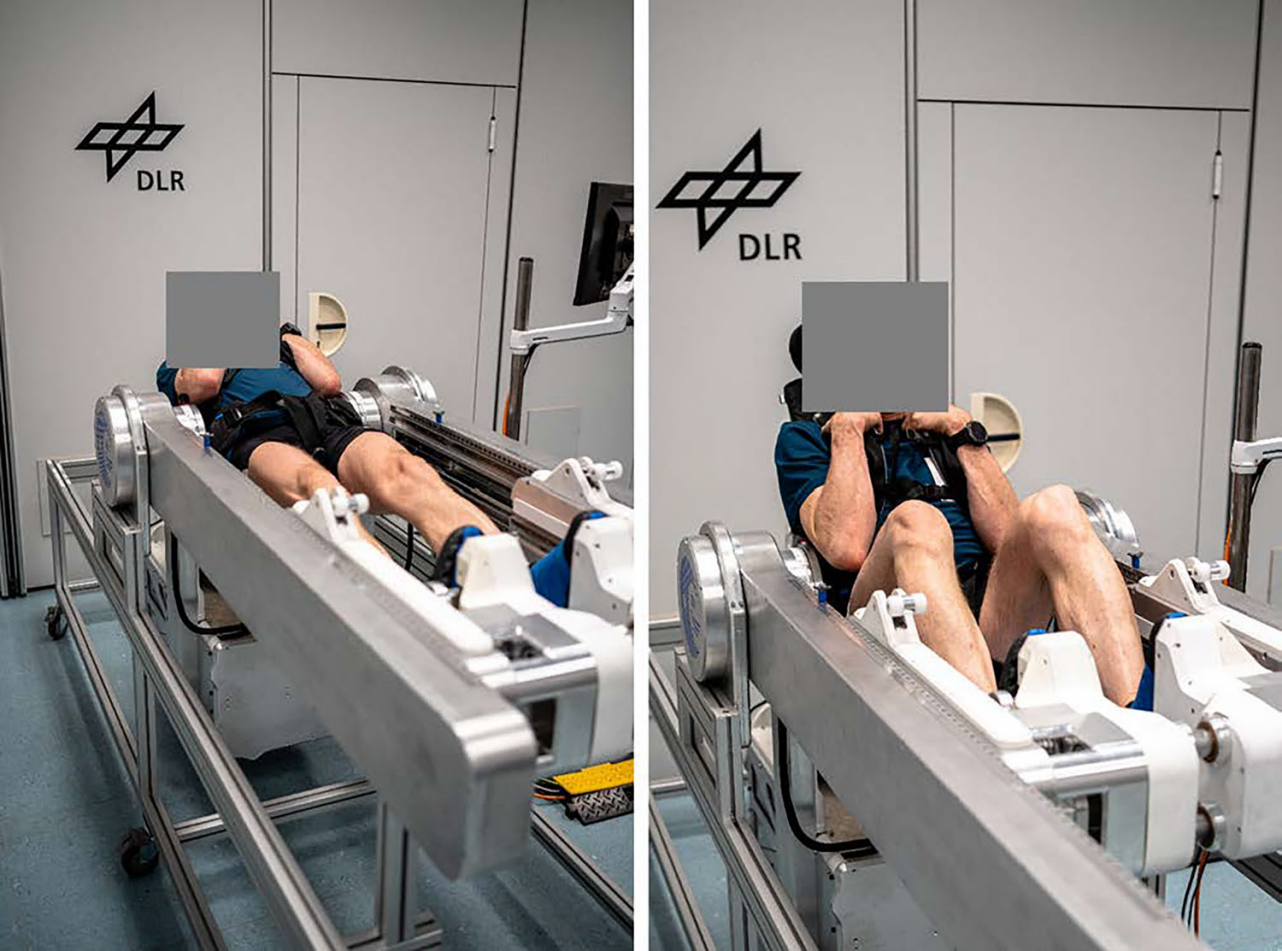
Squats on ATHLETIC by a participant. Left: horizontal, extended position, which is the starting position for a squat. Right: flexion of the knee and hip joints to perform a squat.

The construction of ATHLETIC allowed an almost natural motion during squats (Figure 3 and Supporting information Videos S1 and S2). During the downwards movement with flexion of the hips and knees, the placement of the axis of rotation under the centre of gravity of the trunk led to an alignment of the centre of gravity at the level of the front feet.

The exercises on ATHLETIC were compared with the corresponding reference exercises by means of the total constant force given in kilopond and as percentage of reference exercise (%RE).

### 2.6 Plyometric exercise: Countermovement jumps, reactive hopping

On ATHLETIC and on the ground participants performed countermovement jumps (CMJ) and reactive hopping (HOP). Plyometric reference exercises were performed on a jumping platform measuring ground reaction forces at a resolution of 800 Hz (Leonardo, Novotec Medical GmbH, Pforzheim, Germany). Using the manufacturer's software, we determined the maximum ground forces (kN), the jumping height (cm), maximum velocity (m/s), maximum power (W) and ground contact time (ms).

On the ATHLETIC device, CMJ was similar to squatting with respect to the alignment of the body's centre of gravity placed over the forefeet. During CMJ, participants fall into a shallow squat position and immediately start knee and hip extension to jump as high as possible. The participants were asked to place their hands on their hips, when jumping on ground, and to grip handles on the chest belt while exercising on ATHLETIC.

Participants were allowed to practice several CMJs on both locations before the first measurement. CMJs on ATHLETIC were first practiced with constant forces lower than 100% BW. If CMJs were correctly performed at a reduced force on ATHLETIC, the constant force was stepwise increased until reaching 100% BW in the optimum case. If, however, on ATHLETIC a participant could not correctly perform CMJ at 100% BW, CMJ was finally tested using the highest possible force (Supporting information Videos S3 and S4).

Reactive forefoot hopping (HOP) was performed with almost fully extended hips and knees. For HOP on the ATHLETIC device, the BTA was fixed in a horizontal position. In this position weight and ground force were elastically transmitted over the muscle chain. However, optimum performance of HOP required a highly adjusted stiffness along the body axis. During reactive hops each bounce took advantage of elastic energy stored in the calves and the Achilles tendons and from excess muscle activation generated in the previous landing phase. This process was time dependent and the total ground contact phase including landing and relaunching must not be longer than about 200 ms. Under both conditions, HOP included a series of about 20 hops.

The same variables as for the reference exercise plus the adjusted constant force replacing body weight were analysed from the records on ATHLETIC using custom written software in R. As for resistive exercises, we finally calculated the %RE value for several variables measured on ATHLETIC.

### 2.7 Recording and analysis of muscle activity by electromyography

Muscle activity was determined by transcutaneous measurement of muscle action potentials using electromyography (EMG) (Noraxon Ultium; Software: MR3 version 3.14.76; Noraxon, Scottsdale, AZ, USA). EMG was recorded from selected leg and trunk muscles: right tibialis anterior muscle as part of the foot dorsiflexors, left and right gastrocnemius lateralis muscles as foot plantarflexors, left and right vastus lateralis muscles representing knee extensors, right biceps femoris as knee flexor, right erector spinae muscle as a back muscle erecting the trunk and right rectus abdominis as an anterior trunk muscle flexing the trunk. The signals were recorded at a frequency of 2000 Hz. In addition, a time synchronization signal coming from the ATHLETIC device was recorded.

During exercise over selected periods of muscle contraction, the amplitude (µV) of the EMG signal was calculated as the root mean square. EMG amplitudes measured during exercise on ATHLETIC were finally normalised on the amplitude of the corresponding values determined by reference exercise (RE) and were finally given as %RE. The analysis of the EMG records was processed using the previously mentioned custom‐made software based on the programming language R‐4.3.2 within RStudio 2023.12.0 Build 369.

### 2.8 Questionnaires

After finishing the physical measurement, the participants filled in a questionnaire focusing on ergonomics and naturalism of the movements using LimeSurvey (version 3.22.2, LimeSurvey GmbH, Hamburg, Germany). The complete questionnaire is available as Supporting information File S1.

### 2.9 Statistics

In this evaluation study, we focused on descriptive statistics by calculating mean values and standard deviation. Variables on mechanical performance and muscle activity during exercise on ATHLETIC were normalised to the corresponding values during reference exercise and were given as %RE. The deviation from 100% was tested for significance by a two‐sided one‐sample Student's *t*‐test. For comparing alterations in muscle activation in physical performance on ATHLETIC, the normalised EMG amplitudes were compared with corresponding normalised force values using a paired two‐sided *t*‐test. We used IBM SPSS Statistics software version 26 (IBM Corp, Armonk, NY, USA). A *P*‐value <0.05 was assumed for statistical significance.

## 3 RESULTS

### 3.1 Resistive training: squatting

The coordination of motion in hips and knees during squatting on ATHLETIC was initially challenging for all participants. Practicing with variable numbers of repetitions was needed to (1) gain proficiency in the horizontal, supine position with constant load at the feet as the replacement for the natural upright standing, and (2) to synchronise the motion of different body segments. It turned out that it is an advantage to start squatting with low constant forces, which were then gradually increased.

Most participants were unable to squat on ATHLETIC against forces equal to reference load of 150% BW. Participants could only work with adjusted constant forces of 72 ± 16 kp corresponding with only 65 ± 8%RE (*P *< 0.001, *d* = −4.149). During exercising, the inertia module and friction altered the ground reaction force measured at the feet in addition to the force generated by the constant force mechanism. The actually measured ground reaction force at the foot plate averaged over the entire period of a squat was 67 ± 17 kp. Peak values of ground reaction force reached 101 ± 18 kp. As we did not measure ground force during the reference exercise, we did not calculate %RE for the ground reaction force.

The normalised EMG amplitudes of the vastus lateralis muscles were reduced to 70 ± 28%RE (*P* = 0.009, *d* = −1.051) on the right side and 79 ± 21%RE (*P* = 0.013, *d* = −0.976) on the left side. Further the EMG amplitude of the co‐contracting biceps femoris muscle was reduced to 47 ± 31%RE (*P* < 0.001, *d* = −1.631, Table 1). This means that the %RE values of the EMG amplitudes of vastus lateralis muscles were not different from the corresponding %RE value of the adjusted constant force (0.179 < *P* < 0.869).

**TABLE 1:** EMG peak amplitudes measured during exercise on ATHLETIC normalised to values determined during the corresponding reference exercise (%RE).

	Given constant force (%RE)	Knee extension		Knee flexion	Ankle stabilization			Trunk motion	
		VL, R (%RE)	VL, L (%RE)	BF, R (%RE)	GL, R (%RE)	GL, L (%RE)	TA, R (%RE)	ES, R (%RE)	RA, R (%RE)
Squatting (mean ± SD)	65 ± 8 *	70 ± 28 *	79 ± 21 *	47 ± 31*	91 ± 34	98 ± 41	109 ± 82	101 ± 143	373 ± 593
Countermovement Jump (mean ± SD)	90 ± 14	77 ± 63	94 ± 76	28 ± 28 *	58 ± 31 *	46 ± 18 *	81 ± 41	48 ± 26 *	132 ± 106
Reactive Hopping (mean ± SD)	89 ± 11 *	62 ± 40 *	68 ± 33 *	20 ± 26 *	100 ± 36	100 ± 23	77 ± 36	180 ± 214	48 ± 59 *

Mean ± standard deviation of all performed exercise are shown. *: Significantly different from 100%RE (*P *< 0.05). BF, biceps femoris muscle; ES, erector spinae muscle (lumbar region); GL, gastrocnemius lateralis muscle; L, left; R, right; RA, rectus abdominis muscle (above belly button); TA, tibialis anterior muscle; VL, vastus lateralis muscle.

In both gastrocnemius lateralis muscles, the right tibialis anterior muscle and the right erector spinae muscle the EMG amplitudes as %RE on ATHLETIC were not significantly different from the amplitudes measured as reference (0.444 < *P* < 0.992, Table 1). The value of rectus abdominis muscle is not meaningful as the EMG amplitudes of this muscle were generally very low during the reference squatting, resulting in high variability when using as a denominator for normalising the EMG amplitudes measured on ATHLETIC.

### 3.2 Plyometric training

#### 3.2.1 Countermovement jumps

The CMJ had to be started with very small forces so that the participants could perform a reasonably coordinated movement. The loads were then gradually increased until the maximum load was reached that enabled CMJ‐like exercise. The pair of inertia modules was then adjusted to sum of masses that corresponded with the simulated weight by the constant force mechanism. However, participants were not able to handle this inertia. Finally, it turned out that the sum mass of inertia had always to be reduced to the lowest possible sum value of 58 kg. Further on, only the load by the CFM was varied for testing the participants’ ability to perform CMJ in a correct motion pattern.

**FIGURE 4:**
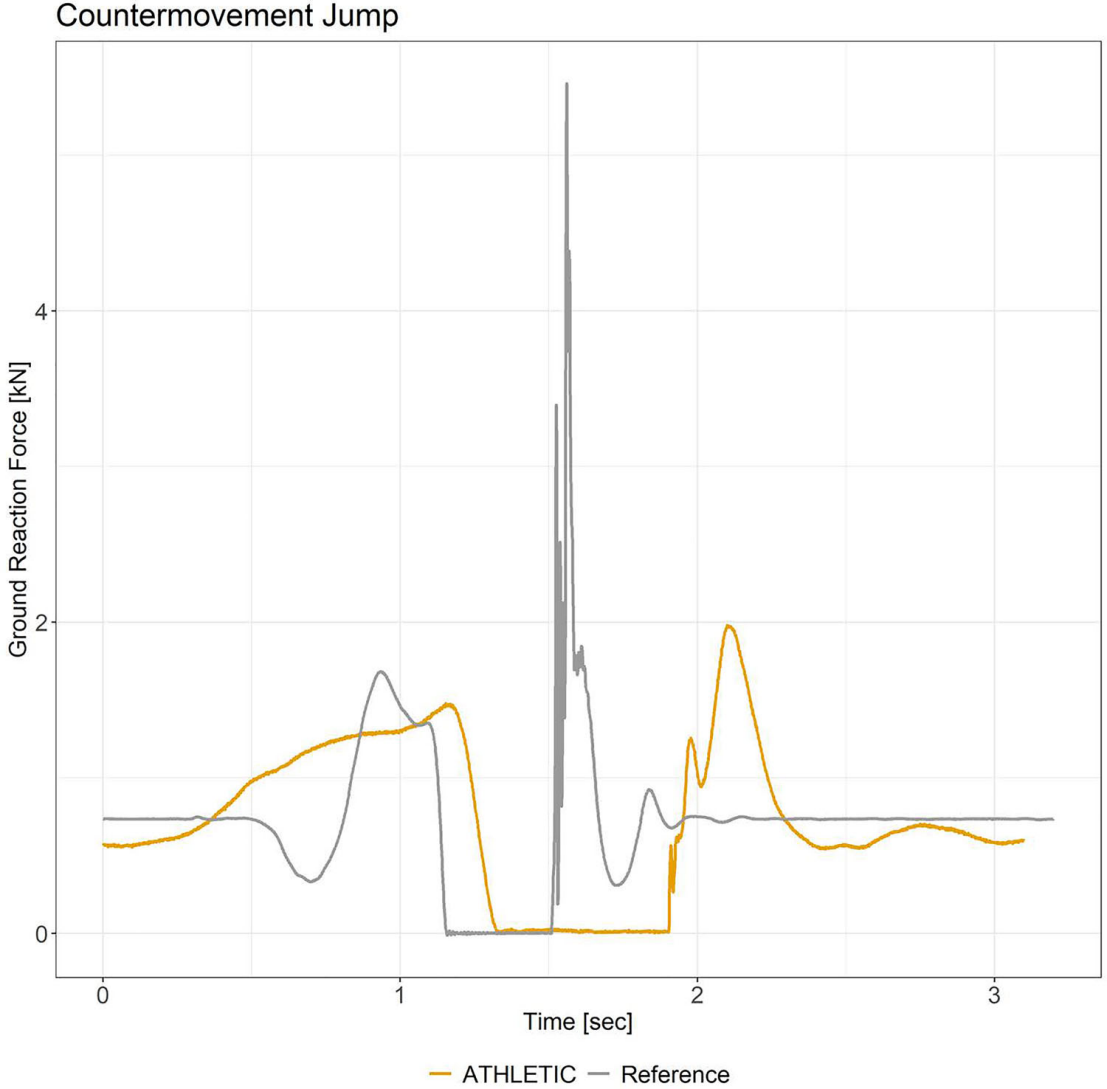
Records of the ground reaction force (GRF) of one participant for countermovement jump (reference in grey and ATHLETIC in yellow). During reference exercise, GRF followed the typical time course including a drop of force before the jump, which was caused by a free‐fall like knee flexion. On ATHLETIC, this drop was not observed because of a slower and more controlled knee flexion. Landing GRF was about 2.5 times higher during reference jumping in the vertical direction for this participant. Additionally, a clearly longer duration for the whole exercise is seen.

Figure 4 shows an example of typical ground reaction force (GRF) traces during CMJ on ATHLETIC and on ground. The CMJ started with a rapid movement from a straight supine posture to a shallow squat. In this initial phase, GRF should drop below body weight on ground or below the given constant force on ATHLETIC, respectively, because of the rapid knee flexion at a velocity close to free fall. On ATHLETIC, however, this initial drop of force was not observed. Likely, the participants involuntarily avoided the rapid, free‐fall like knee flexion and performed a slower and more controlled motion instead. Afterwards, the phase of breaking the fall and launching of the jump should merge into one action, in order to utilize the reflex activation of stretched knee extensor muscles and the elastically stored energy in these muscles and the patellar tendon for the following jump. Merging these two actions should ideally result in one steep peak of the GRF before launch, followed by the flight phase, where GRF is zero, and during landing on ground in a steep high force peak.

However, on ATHLETIC landing typically was much softer than on ground. During the flight phase, participants remained in somewhat flexed posture of hips and knees, which resulted in a less stiff landing as indicated by GRF showing a much lower peak amplitude and a broader and often split peak with sometimes even some swinging.

During CMJ on ATHLETIC, participants could jump against a constant force of 90 ± 14%RE, which was not significantly lower than their body weight serving as reference load (*P* = 0.059, Table 2). However, CMJ performance on ATHLETIC was much inferior than on ground. The peak velocity in the launch phase reached only 18 ± 7%RE (*P* < 0.001, *d* = −11.384) and in consequence the jumping height on ATHLETIC was only 25 ± 13%RE (*P* < 0.001, *d* = −5.930, Table 2).

**TABLE 2:** Results of the analysis of the mechanical characteristics of plyometric exercises on ATHLETIC.

	Given constant force (%RE)	Peak ground force (%RE)	Jumping height (%RE)	Maximum speed (%RE)	Maximum power (%RE)	Mean ground contact time (ms)	Mean ground contact time (%RE)
Countermovement jump (mean ± SD)	90 ± 14	90 ± 16	25 ± 13 *	18 ± 7 *	3 ± 3 *	—	—
Reactive Hopping (mean ± SD)	89 ± 11 *	47 ± 7 *	21 ± 12 *	—	—	620 ± 110	357 ± 38 *

Mean and standard deviation (SD) of countermovement jump and reactive hopping are shown. Given constant force is the applied load by ATHLETIC compared to the reference exercise in vertical direction (in %RE: percentage load of reference testing). Peak ground force during launching, maximum jumping height, maximum speed, and maximum power in relation to reference testing are given as %RE. Mean ground contact time (ms) is the mean absolute time on ground between two reactive hops in the series of about 20 hops. Mean ground contact time in relation to mean ground contact during reference testing is given as %RE. *Significant difference compared to reference testing with *P *< 0.05.

During jumping on ATHLETIC, peak amplitudes of the vastus lateralis muscle were very variable and on average not significantly lower than on ground (on the right side: 77% ± 63%RE, *P* = 0.279; on the left side: 94 ± 76%RE, *P* = 0.800, Table 1) The peak amplitudes of the right biceps femoris muscles during CMJ on ATHLETIC were also very variable and on average much lower than on ground (28 ± 28%RE, *P* < 0.001, *d* = −2.540, Table 1).

In the lower legs, peak EMG amplitudes of gastrocnemius lateralis muscles were significantly lower during CMJ on ATHLETIC than on ground (right side: 46 ± 18%RE, *P* = 0.002, *d* = −1.353, left side: 58 ± 31%RE, *P *< 0.001, *d* = −2.939). The EMG peak amplitude of the right tibialis anterior muscle, however, was not significantly different (81 ± 41%RE, *P* = 0.212, Table 1).

The trunk muscle erector spinae showed significantly lower EMG amplitudes during CMJ on ATHLETIC than on ground (48 ± 26%RE, *P *< 0.001, *d* = −2.048, Table 1). The normalised values of the rectus abdominis values were again of little meaning, because their EMG showed little activity during CMJ on ground, similar to the situation during squatting.

#### 3.2.2 Reactive hopping

When loaded with a constant force corresponding with their body weight (100%RE), all participants could perform a rapid plantarflexion of their feet that resulted in a separation of their feet from the platform. However, participants were not able to perform a reactive forefoot hopping (HOP) at that load. Higher loads and too low loads worsen the performance. We had to find individualised optimum loads, at which the performance of jumping came as close to well performed HOP as possible. Finally, the mean given constant force during HOP was adjusted to 89 ± 11%RE (*P* = 0.034, *d* = −0.697, Table 2).

**FIGURE 5:**
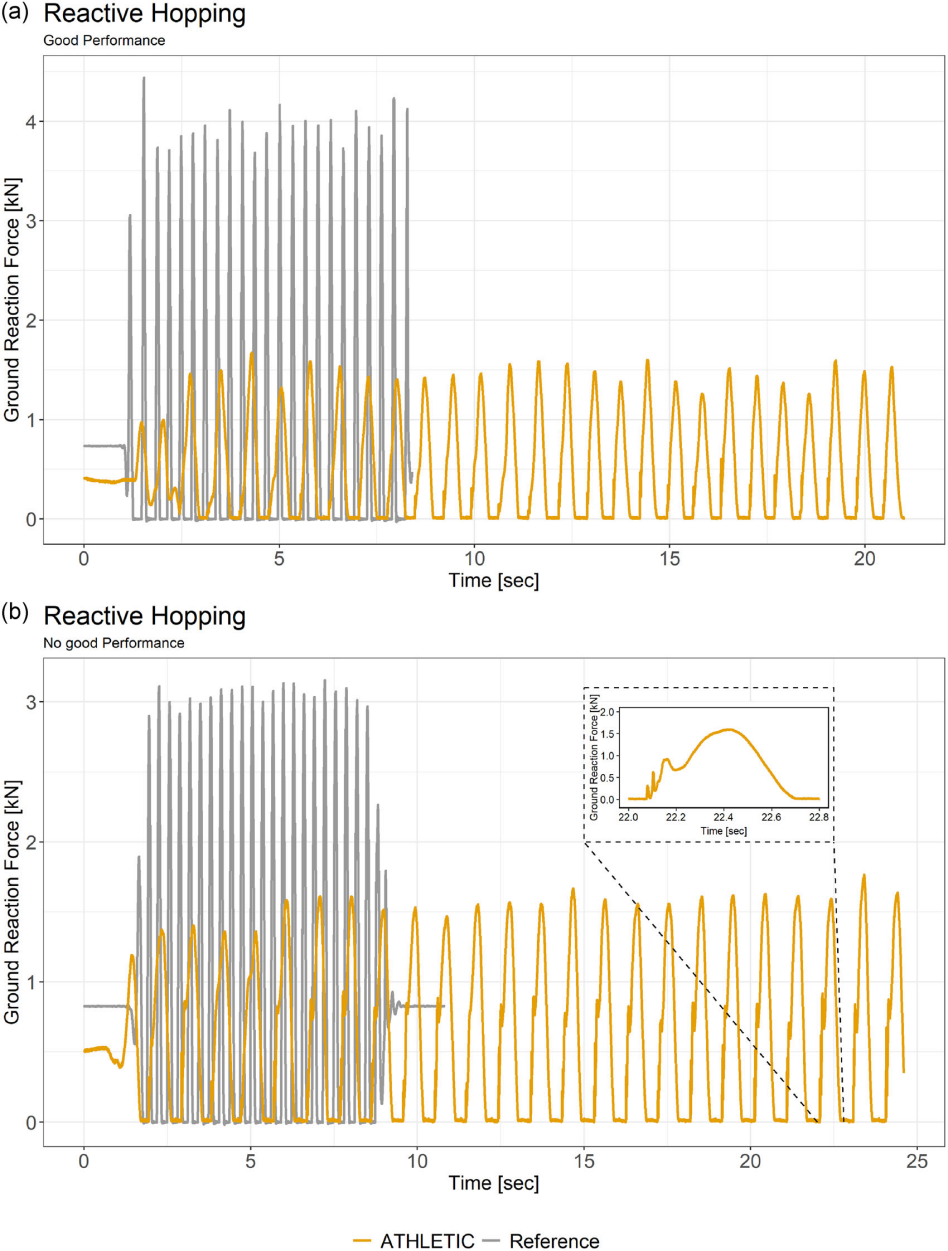
Records of the ground reaction force (GRF) of two participants for reactive hopping (reference in grey and ATHLETIC in yellow). (a) The GRF during reactive hopping of a participant, who performed the reactive hopping during exercising on ATHLETIC quite well. (b) The performance of a participant, who was not used to reactive hopping. The reactive hopping in the vertical direction (reference) shows one peak of GRF, but during hopping on ATHLETIC there were two peaks (one smaller and one higher peak), which shows that there was an active landing phase and an active take‐off phase (no longer a reactive hopping). In total, the time for performing about 20 hops was clearly longer for hopping on ATHLETIC (for both participants).

Figure 5 shows typical records of GRFs during HOP on ATHLETIC and on ground. The inter‐individual performance on ATHLETIC was variable across all participants. Figure 5a shows an example of nearly well performed HOP on ATHLETIC with a merged GRF for landing and relaunching during ground contact between single hops. Figure 5b shows a worse performance with a split landing and relaunching phase.

Average values shown in Table 2 underline the poor performance of reactive hopping on ATHLETIC in comparison with the performance on ground. Peak ground forces (47 ± 7%RE, *P* < 0.001, *d* = −4.314) and the jumping height (21 ± 12%RE, *P* < 0.001, *d* = −7.125) were both distinctly worse. Moreover, ground contact time was far above the given maximum of 200 ms (620 ± 110 ms) and far longer than ground contact times during HOP on ground (357 ± 38%RE, *P* < 0.001, *d* = 2.347). In conclusion, HOP on ATHLETIC was not the expected reactive exercise.

EMG peak amplitudes of the gastrocnemius were not altered with respect to values at HOP on ground (right side: 100 ± 36%RE, *P* = 0.975; left side:100 ± 23%RE, *P* = 0.956).

Significantly lower EMG peak amplitudes during HOP on ATHLETIC were found in the vastus lateralis muscle (right side: 62 ± 40%RE, *P *< 0.014, *d* = −0.961; left side: 68 ± 33%RE, *P* = 0.012, *d* = −0.994) and, moreover in the biceps femoris muscle (20 ± 26%RE, *P* < 0.001, *d* = −3.107, Table 1).

During HOP on ATHLETIC the right erector spinae muscle showed very variable values of EMG peak amplitudes with no significant difference from reference (183 ± 215%RE, *P* = 0.295) whereas the rectus abdominis muscle showed reduced amplitudes (48 ± 59%RE, *P* = 0.022, *d* = −0.874, Table 1).

### 3.3 Questionnaire – ergonomics and movement

The results of the questionnaire on comfort, ergonomics and naturalism of movement showed the following average results (individual results in Table 3). In the lying, horizontal position, comfort was rated 4.5 out of a maximum of 5 points, while the seated position with 90 degrees at the hip angle was rated 4 (less comfortable). In relation to the shoulder pad, which together with the hip belt transferred the load to the user, comfort, fitting, support and pressure were each rated 4. For the hip belt, comfort and fitting were rated at 4.5, support at 4 and pressure at 2. Other aspects were comfort (4), fitting (4), support (4.5) and sweat accumulation (3) when using the shoes that connect the user's feet to the device. Finally, the naturalism of movement in the hip joint was rated at 3, in the knee joint at 4 and in the ankle joint at 3 (Table 3).

**TABLE 3:** Results of the questionnaire about ergonomics, comfort and naturalism of the movements.

Category		Scoring					Median
		1	2	3	4	5	
Lying position	Comfort	0	0	1	4	5	4.5
Sitting position	Comfort	0	0	3	5	2	4
Shoulder pad	Comfort	0	0	2	6	2	4
	Fitting	0	1	0	7	2	4
	Hold	0	0	2	6	2	4
	Pressure	0	0	4	4	2	4
Hip belt	Comfort	0	0	1	4	5	4.5
	Fitting	0	0	1	4	5	4.5
	Support	0	1	3	2	4	4
	Pressure	1	5	4	0	0	2
Shoes	Comfort	0	0	2	4	4	4
	Fitting	0	0	3	3	4	4
	Support	0	0	2	3	5	4.5
	Sweat Accumulation	1	0	5	3	1	3
Movement naturalism	Hip Joint	0	3	3	3	1	3
	Knee Joint	0	1	2	5	2	4
	Ankle Joint	0	3	3	4	0	3

The table shows the number of participants, which rated the specific topic/category. The scoring was as follows. Comfort: 1 = uncomfortable, 2 = less uncomfortable, 3 = neutral, 4 = less comfortable, 5 = comfortable. Fitting/hold/support: 1 = not good, 2 = less good, 3 = neutral, 4 = good, 5 = very good. Pressure/sweat accumulation: 1 = very weak, 2 = weak, 3 = neutral, 4 = strong, 5 = very strong. Movement naturalism: 1 = not natural, 2 = less natural, 3 = neutral, 4 = natural, 5 = very natural.

## 4 DISCUSSION

Preliminary tests of this study have shown that ATHLETIC is suitable for intensive strength training such as leg press exercise performed with a fixed trunk support at 80°. In this fixed body position, a person can train with forces up to the maximum adjustable constant force of 300 kp. Therefore, the device was tested in this study for exercise modes involving coordinatively challenging movements in the hip and knee joints (squat, CMJ), as well as for reactive muscle activation and the storage and return of energy to muscles and tendons during plyometric exercises (CMJ, HOP).

In both, squats and CMJ, the body is bent at the hips and knees so that the centre of mass of the upper body is above the forefoot, thus keeping the body in balance and involving the entire muscle chain from the torso to the feet in the exercise. This movement sequence is enforced on ATHLETIC by placing the support for the trunk in the centre of gravity of the upper body and this axis of rotation is always at the height of the forefoot.

After an initial familiarisation period, squatting was possible, but with reduced loads compared to the reference exercises (65 ± 8%RE) and reduced muscle activation in the knee extensors. For this exercise mode, the constant force was adjusted while the participants were unloaded by a knee flexion to a mechanical end stop. The participant started squatting with a knee extension from this deep squat. However, a too small knee angle was not the main reason for the lower training weight on ATHLETIC, as the participants were helped with this first stretch. The coordination of squatting and all other modes of exercise on ATHLETIC was disturbed by the presence of gravity. Being carried by the device and loaded by the constant force mechanism in a horizontal, lying position as equivalent to upright standing was quite challenging. The participants were therefore unable to use their involuntary motor programmes with which they normally coordinate the exercises on the floor. The fact that during squatting the muscle activation decreased to the same extent as the peak amplitudes of the EMG of the knee extensors emphasises the impression that the participants were too uncertain to work with the reference force. But on the other hand, the origin of reduced EMG peak amplitudes could be the general design of the ATHLETIC device as this allows no natural movement.

The uncertainty and lack of coordination of movement due to the unfamiliar body position and the disturbing weight force perpendicular to the longitudinal axis of the body became even more apparent in the CMJs. On average, the participants trained with 90 ± 14%RE during CMJ, which did not significantly differ from reference. This was also reflected in the maximum amplitudes of muscle activation of the knee extensors, which also did not differ from the reference measurement (Table 1). However, despite comparable muscle strength, the participants only achieved a low jumping speed and therefore low jumping height. This again indicates that due to poor muscle coordination and unfamiliar exercise execution, for example, the missing free falling during the countermovement, the movement sequence was not optimal. As for squatting, the unfamiliar horizontal position as well as the general design of the device could be the origin of the reduced results compared to reference exercising.

At the end of a CMJ, the participants did not end up in a stiff, largely stretched posture, but with slightly bent knees and hip joints. In addition to the involuntary fear of landing too hard after the jump, the uncertainty about the time of landing also contributed to this soft protective posture. The majority of the participants could hardly be prevented from looking at their feet when landing resulting in a bent posture. None of the participants had this issue during the reference jumps on ground. The inertia set on the device had to be adjusted to the minimum value for all participants so that they could move approximately normally during the CMJ. At higher inertia values, the movement was perceived as too tough and slow. This meant that the set inertial mass was always smaller than the heavy mass set by the constant force mechanism, which differs to the normal situation when jumping on Earth. It is possible that higher forces than calculated were generated in the inertial mechanism during the fast movements in the jump. The very low friction in the system could also play a role. Overall, however, the relationship between gravity and inertia in the CMJ on ATHLETIC needs to be further investigated.

Reactive forefoot hopping needed a reduction of the constant force to 89 ± 11% BW and the inertia module remained at its adjustable minimum. None of the participants reached the hopping heights and short ground contact times that are characteristic of hopping and were achieved by all participants when hopping on the ground (Table 2, Figure 5). Participants who also regularly perform plyometric training such as hopping in their everyday training showed shorter ground contact times (Figure 5a) than participants who did not profit from regular plyometric training (Figure 5b). However, the target ground contact time of 200 ms or less was not achieved by any of the participants.

The poor performance of reactive hopping could be explained by an insufficient stiffness along the body axis. This conclusion was supported by the low EMG amplitudes in muscles stabilising the knee, hip and trunk. One reason for the lack of stiffness could be the unloading of the upper body being carried by the back‐support system (Piotrowski et al., 2018). Another reason could be an inadequate hold by the backpack system. The belts placed over the shoulder were too soft when counteracting the ground force in upright posture and sometimes the mechanism used for the individual length adjustment even gave way for some millimetres. Further tests need to prove whether coordination can be improved through further practice and familiarisation to the odd posture so that jumping on ATHLETIC can be as efficient as on the ground. Experience with a previously tested horizontal training device, in which the upper body was pulled against a base plate while lying on a sled using a pair of vacuum springs, has shown that most participants require several training sessions to achieve an optimal sequence of movements during plyometric exercises (Kramer et al., 2017).

The results of the questionnaire showed that the device is very comfortable both when seated and when lying down. The load transfer could be optimised, especially in the pelvic area to relieve the shoulders. Currently, the majority of the force was applied at the shoulders, which can lead to pain, friction and possibly skin irritation during prolonged training. As almost daily training is necessary in a microgravity environment, comfort is a factor that can determine the success of a countermeasure maintaining musculoskeletal health. In addition to comfort, however, it is very important that the movements on the device feel natural. The naturalness of the movement was rated as natural in the knee joint in particular, and still neutral in the hip and ankle joints.

As the aim of this study only was to test the concept of an exoskeleton‐like device, it needs to be mentioned that this prototype is neither weight‐ nor size‐optimised for spaceflight.

In conclusion, the design of ATHLETIC enables a natural movement sequence for exercises such as squats, countermovement jumps or forefoot hopping in the perception of the subjects. However, the performance achieved and the quality of execution of the exercises were significantly inferior to reference exercises on the ground. Lying on the device, which not only guided the movement sequences but also carried the weight of the participants, did not allow the participants to simply use the motor programmes for the exercises on the ground. Additionally, the design of the device could restrict the performances. Thus, a further study is currently planned to show whether only by practicing for several days the plyometric jumps on the ATHLETIC the participants can improve the movement sequence to such an extent that these jumps reach the quality of execution and the mechanical performance coming close to natural jumps on the ground. In addition, improvements in the fixation of the participants are to be tested in the follow‐up study. Finally, further tests must examine the training behaviour of people in weightlessness, where the ATHLETIC holding elements only guide the participants in their movements and no longer support them.

## AUTHOR CONTRIBUTIONS

Jonas Böcker, Jochen Zange and Jörn Rittweger contributed to the design and implementation of the study. Jonas Böcker, Jochen Zange, Jörn Rittweger, Guillaume Fau, Torsten Siedel and Sebastian Langner developed the countermeasure device. Jonas Böcker, Jochen Zange and Guillaume Fau wrote the manuscript. All authors revised and corrected the manuscript before submission. All authors have read and approved the final version of this manuscript and agree to be accountable for all aspects of the work in ensuring that questions related to the accuracy or integrity of any part of the work are appropriately investigated and resolved. All persons designated as authors qualify for authorship, and all those who qualify for authorship are listed.

## CONFLICT OF INTEREST

The ATHLETIC project was funded by ESA and ESA was informed regularly about the project progress. Thus, ESA gave their feedback regarding the planned study, but was not involved in the conduction of the study or the analysis of the results. Space Applications Services manufactured the device and wrote parts of this manuscript, but was not involved in planning and conducting the study, as well as analysing the results or formulate the conclusions. deuter Sport GmbH provided the harness for transferring the load of the device to the subject, but did not participate in conducting the study or analysing the results.

## Supporting information



File S1. Questionnaire.

Video S1. Squatting 1.

Video S2. Squatting 2.

Video S3. Jumping 1.

Video S4. Jumping 2.

## Data Availability

The data are stored locally at the German Aerospace Center. The data can be made available on request with appropriate justification and approval by ESA.
