# Supplementary material for: ATHLETIC: An exoskeleton countermeasure exercise device for resistive and plyometric training in deep‐space missions
Source: Exp Physiol. 2025 Mar 20;111(9):4153–65. doi: 10.1113/EP092263 (PMC13394484; doi:10.1113/EP092263)
Supplement: Supplementary file 1 [file EPH-111-4153-s002.pdf]

Athletic: Fragebogen zum Komfort und zur Ergonomie

Bitte beantworten Sie durch Ankreuzen die folgenden Fragen zum Komfort und zur Ergonomie des Athletic-Gerätes.

Komfort im Sitzen

1) Wie bequem ist das Gerät bei Sitzen mit aufgerichtetem Körper?

gar nicht bequem						sehr bequem
	1	2	3	4	5	

Komfort im Liegen

2) Wie bequem ist das Gerät beim Liegen mit gestrecktem Körper?

gar nicht bequem						sehr bequem
	1	2	3	4	5	

Schulterpolster

3) Wie bequem ist das Schulterpolster?

gar nicht bequem						sehr bequem
	1	2	3	4	5	

4) Wie gut ist die Passform des Schulterpolsters?

gar nicht gut						sehr gut
	1	2	3	4	5	

5) Wie gut ist der Halt den das Schulterpolster bietet?

gar nicht gut						sehr gut
	1	2	3	4	5	

6) Wie stark ist der lokale Druck am Schulterpolster, den sie gespürt haben?

sehr schwach						sehr stark
	1	2	3	4	5	

Beckengurte

7) Wie bequem sind die Beckengurte?

gar nicht bequem						sehr bequem
	1	2	3	4	5	

8) Wie gut ist die Passform der Beckengurte?

gar nicht gut						sehr gut
	1	2	3	4	5	

9) Wie gut ist der Halt den die Beckengurte bieten?

gar nicht gut						sehr gut
	1	2	3	4	5	

10) Wie stark ist der lokale Druck an den Beckengurten, den sie gespürt haben?

sehr schwach						sehr stark
	1	2	3	4	5	

Schuhe

11) Wie bequem sind die Schuhe?

gar nicht bequem						sehr bequem
	1	2	3	4	5	

12) Wie gut ist die Passform der Schuhe?

gar nicht gut						sehr gut
	1	2	3	4	5	

13) Wie gut ist der Halt den die Schuhe bieten?

gar nicht gut						sehr gut
	1	2	3	4	5	

14) Wie stark war die Schweißansammlung an den Füßen?

gar nicht						sehr stark
	1	2	3	4	5	

Bewegung

1) Wie natürlich empfanden Sie die Bewegung im <u>Hüftgelenk</u>?

gar nicht natürlich						sehr natürlich
	1	2	3	4	5	

1) Wie natürlich empfanden Sie die Bewegung im <u>Kniegelenk</u>?

gar nicht natürlich						sehr natürlich
	1	2	3	4	5	

1) Wie natürlich empfanden Sie die Bewegung im <u>Sprunggelenk</u>?

gar nicht natürlich						sehr natürlich

Falls Probleme auftraten, können Sie diese bitte schildern? Bitte nehmen Sie dabei möglichst Bezug auf die Körperposition und die Bauteile, welche die Probleme hervorgerufen haben.

____

____

____

____
